# A global database of soil nematode abundance and functional group composition

**DOI:** 10.1038/s41597-020-0437-3

**Published:** 2020-03-26

**Authors:** Johan van den Hoogen, Stefan Geisen, Diana H. Wall, David A. Wardle, Walter Traunspurger, Ron G. M. de Goede, Byron J. Adams, Wasim Ahmad, Howard Ferris, Richard D. Bardgett, Michael Bonkowski, Raquel Campos-Herrera, Juvenil E. Cares, Tancredi Caruso, Larissa de Brito Caixeta, Xiaoyun Chen, Sofia R. Costa, Rachel Creamer, José Mauro da Cunha e Castro, Marie Dam, Djibril Djigal, Miguel Escuer, Bryan S. Griffiths, Carmen Gutiérrez, Karin Hohberg, Daria Kalinkina, Paul Kardol, Alan Kergunteuil, Gerard Korthals, Valentyna Krashevska, Alexey A. Kudrin, Qi Li, Wenju Liang, Matthew Magilton, Mariette Marais, José Antonio Rodríguez Martín, Elizaveta Matveeva, El Hassan Mayad, E. Mzough, Christian Mulder, Peter Mullin, Roy Neilson, T. A. Duong Nguyen, Uffe N. Nielsen, Hiroaki Okada, Juan Emilio Palomares Rius, Kaiwen Pan, Vlada Peneva, Loïc Pellissier, Julio Carlos Pereira da Silva, Camille Pitteloud, Thomas O. Powers, Kirsten Powers, Casper W. Quist, Sergio Rasmann, Sara Sánchez Moreno, Stefan Scheu, Heikki Setälä, Anna Sushchuk, Alexei V. Tiunov, Jean Trap, Mette Vestergård, Cecile Villenave, Lieven Waeyenberge, Rutger A. Wilschut, Daniel G. Wright, Aidan M. Keith, Jiue-in Yang, Olaf Schmidt, R. Bouharroud, Z. Ferji, Wim H. van der Putten, Devin Routh, Thomas W. Crowther

**Affiliations:** 1 Department of Environmental Systems Science, Institute of Integrative Biology, ETH Zürich, Zürich, Switzerland; 2 Department of Terrestrial Ecology, Netherlands Institute of Ecology, Wageningen, The Netherlands; 3 Department of Biology and School of Global Environmental Sustainability, Colorado State University, Fort Collins, CO USA; 4 Asian School of the Environment, Nanyang Technological University, Singapore, Singapore; 5 Animal Ecology, Bielefeld University, Bielefeld, Germany; 6 Soil Biology Group, Wageningen University & Research, Wageningen, The Netherlands; 7 Department of Biology, Evolutionary Ecology Laboratories, Monte L. Bean Museum, Brigham Young University, Provo, UT USA; 8 Nematode Biodiversity Research Laboratory, Department of Zoology, Aligarh Muslim University, Aligarh, India; 9 Department of Entomology & Nematology, University of California, Davis, CA USA; 10 Department of Earth and Environmental Sciences, The University of Manchester, Manchester, UK; 11 Institute of Zoology, Terrestrial Ecology, University of Cologne and Cluster of Excellence on Plant Sciences (CEPLAS), Cologne, Germany; 12 Instituto de Ciencias de la Vid y del Vino (Universidad de La Rioja, CSIC, Gobierno de La Rioja), Logroño, Spain; 13 Department of Phytopathology, Institute of Biological Sciences, University of Brasília, Brasília, Brazil; 14 School of Biology and Environmental Science, University College Dublin, Belfield, Dublin 4 Ireland; 15 Soil Ecology Laboratory, College of Resources and Environmental Sciences, Nanjing Agricultural University, Nanjing, China; 16 Centre of Molecular and Environmental Biology, University of Minho, Braga, Portugal; 17 Empresa Brasileira de Pesquisa Agropecuária (Embrapa), Centro de Pesquisa Agropecuária do Trópico Semiárido, Petrolina, Brazil; 18 Zealand Institute of Business and Technology, Slagelse, Denmark; 19 Institut Sénégalais de Recherches Agricoles/CDH, Dakar, Senegal; 20 Instituto de Ciencias Agrarias, CSIC, Madrid, Spain; 21 Crop and Soil Systems Research Group, SRUC, Edinburgh, UK; 22 Senckenberg Museum of Natural History Görlitz, Görlitz, Germany; 23 Institute of Biology of Karelian Research Centre, Russian Academy of Sciences, Petrozavodsk, Russia; 24 Department of Forest Ecology and Management, Swedish University of Agricultural Sciences, Umeå, Sweden; 25 Laboratory of Functional Ecology, Institute of Biology, University of Neuchâtel, Neuchâtel, Switzerland; 26 J. F. Blumenbach Institute of Zoology and Anthropology, University of Göttingen, Göttingen, Germany; 27 Institute of Biology of the Komi Scientific Centre, Ural Branch of the Russian Academy of Sciences, Syktyvkar, Russia; 28 Erguna Forest-Steppe Ecotone Research Station, Institute of Applied Ecology, Chinese Academy of Sciences, Shenyang, China; 29 School of Biological Sciences, Institute for Global Food Security, Queen’s University of Belfast, Belfast, UK; 30 Nematology Unit, Agricultural Research Council, Plant Health and Protection, Pretoria, South Africa; 31 Department of Environment, Instituto Nacional de Investigación y Tecnología Agraria y Alimentaria, Madrid, Spain; 32 Laboratory of Biotechnology and Valorization of Natural Resources, Faculty of Science Agadir, Ibn Zohr University, Agadir, Morocco; 33 Department of Biological, Geological and Environmental Sciences, University of Catania, Catania, Italy; 34 Department of Plant Pathology, University of Nebraska-Lincoln, Lincoln, NE USA; 35 Ecological Sciences, The James Hutton Institute, Dundee, UK; 36 Institute of Ecology and Biological Resources, Vietnam Academy of Science and Technology, Hanoi, Vietnam; 37 Hawkesbury Institute for the Environment, Western Sydney University, Penrith, New South Wales Australia; 38 Nematode Management Group, Division of Applied Entomology and Zoology, Central Region Agricultural Research Center, NARO, Tsukuba, Japan; 39 Institute for Sustainable Agriculture, Spanish National Research Council, Córdoba, Spain; 40 Ecological Processes and Biodiversity, Center for Ecological Studies, Chengdu Institute of Biology, Chinese Academy of Sciences, Chengdu, China; 41 Institute of Biodiversity and Ecosystem Research, Bulgarian Academy of Sciences, Sofia, Bulgaria; 42 Landscape Ecology, Institute of Terrestrial Ecosystems, Department of Environmental Systems Science, ETH Zürich, Zürich, Switzerland; 43 Swiss Federal Research Institute WSL, Birmensdorf, Switzerland; 44 Department of Phytosanitary Defense, Universidade Federal de Santa Maria, Santa Maria, RS Brazil; 45 Biosystematics Group, Wageningen University, Wageningen, The Netherlands; 46 Laboratory of Nematology, Wageningen University, Wageningen, The Netherlands; 47 Institute of Biology, University of Neuchâtel, Neuchâtel, Switzerland; 48 Plant Protection Products Unit, Instituto Nacional de Investigación y Tecnología Agraria y Alimentaria, Madrid, Spain; 49 Centre of Biodiversity and Sustainable Land Use, University of Göttingen, Göttingen, Germany; 50 Faculty of Biological and Environmental Sciences, Ecosystems and Environment Research Programme, University of Helsinki, Lahti, Finland; 51 A. N. Severtsov Institute of Ecology and Evolution, Russian Academy of Sciences, Moscow, Russia; 52 Eco&Sols, University of Montpellier, CIRAD, INRA, IRD, Montpellier SupAgro, Montpellier, France; 53 Department of Agroecology, AU-Flakkebjerg, Aarhus University, Slagelse, Denmark; 54 ELISOL Environnement, Congénies, France; 55 Plant Sciences Unit, Flanders Research Institute for Agriculture, Fisheries and Food, Merelbeke, Belgium; 56 UK Centre for Ecology & Hydrology, Lancaster Environment Centre, Lancaster, UK; 57 Department of Plant Pathology and Microbiology, National Taiwan University, Taipei, Taiwan; 58 UCD School of Agriculture and Food Science, University College Dublin, Belfield, Dublin 4 Ireland; 59 Research Unit of Integrated Crop Production, Centre Regional de la Recherche Agronomique d’Agadir, Agadir, Morocco; 60 Institut Agronomique et Vétérinaire Hassan II, Campus d’Agadir, Département de Protection des Plantes, Agadir, Morocco

**Keywords:** Biogeography, Biodiversity, Ecosystem ecology

## Abstract

As the most abundant animals on earth, nematodes are a dominant component of the soil community. They play critical roles in regulating biogeochemical cycles and vegetation dynamics within and across landscapes and are an indicator of soil biological activity. Here, we present a comprehensive global dataset of soil nematode abundance and functional group composition. This dataset includes 6,825 georeferenced soil samples from all continents and biomes. For geospatial mapping purposes these samples are aggregated into 1,933 unique 1-km pixels, each of which is linked to 73 global environmental covariate data layers. Altogether, this dataset can help to gain insight into the spatial distribution patterns of soil nematode abundance and community composition, and the environmental drivers shaping these patterns.

## Abstract (summary)

**Table:** 

Measurement(s)	Abundance • Nematoda • environmental factor
Technology Type(s)	Elutriative Centrifugation • computational modeling technique
Factor Type(s)	geographic location
Sample Characteristic - Organism	Nematoda
Sample Characteristic - Environment	soil environment • climate
Sample Characteristic - Location	Earth (planet)

Machine-accessible metadata file describing the reported data: 10.6084/m9.figshare.11925843

## Background & Summary

To generate a global and quantitative understanding of the biogeography of soil organisms, critical players in global biogeochemistry, large and comprehensive datasets are needed. Due to methodological challenges and the labor-intensiveness of characterizing soil biota, many previous studies have focused on a relatively limited number of spatially distinct sampling sites. Whilst these studies are valuable to dissect local and regional scale patterns, they may not hold the depth of information that is needed to feed global-scale models^1^.

Soil nematodes are present in all trophic levels in the soil food web, play central roles in regulating carbon and nutrient dynamics, control soil microorganism populations^2–4^ and, consequently, are good indicators of biological activity in soils^5^. Here, we present a dataset of 6,825 spatially distinct soil nematode samples from all terrestrial biomes and continents, an updated version of the dataset that was originally used to create a global map of soil nematode abundance and community composition^6^. The original version contained 6,759 samples; the updated version that we present here contains 66 additional samples located in Ireland. This dataset can prove useful to disentangle the effects of environmental drivers of soil nematode abundance and community composition across broad spatial scales. The original version of this dataset was used to create a high-resolution map of soil nematode abundance, which revealed that nematodes are present in higher densities in sub-Arctic regions compared to tropical and temperate regions^6^. Soil properties are the primary drivers of soil nematode abundance, whereas climatic conditions have an indirect effect by altering soil conditions^6^. The overall latitudinal gradient, with decreasing abundance towards the equator, is the inverse of patterns often observed in aboveground organisms, but is in line with what has been shown for other belowground biota^7,8^.

Besides data on the total number of nematodes per sample, the dataset contains quantification of the abundance of individuals in different functional groups of soil nematodes classified according to five feeding guilds^9^: bacterivores, fungivores, herbivores, omnivores, predators. For geospatial mapping, these sampling data were aggregated into 1,933 unique 30 Arc-seconds pixels (~1 km^2^ at the equator) and combined with 73 global covariate layers including information on soil physiochemical properties, and vegetation, climate, and topographic, anthropogenic, and spectral reflectance information. We intend to continue expanding the dataset and are open to contributions of additional data.

## Methods

### Data collection

**Fig. 1:**
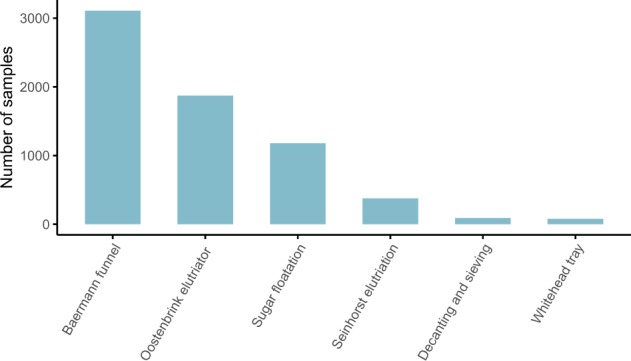
Nematode extraction methods used. The majority of the samples were processed using the Baermann funnel method and Oostenbrink elutriation.

The methods described here are expanded versions of descriptions in our related work^6^. The dataset encompasses georeferenced data on soil nematode abundances according to trophic groups, which were assigned according to Yeates *et al*.^9^. In total, the dataset contains 6,825 georeferenced samples collected in the top 15 cm of soils, including 66 additional samples compared to the dataset used in our related work^6^. Across all samples, 67.2% originate from natural sites and 32.8% from agricultural or managed sites. Nematodes were extracted from soil using standard elutriation methods, including the Baermann funnel method^10^, sugar-floatation/centrifugation^11,12^, decanting and sieving^13^, Oostenbrink elutriation^14^, Whitehead tray^15^ and Seinhorst elutriation^16^. These methods may include variations of the original methods. Most samples present in the dataset were obtained using the Baermann funnel method, followed by Oostenbrink elutriation and sugar-flotation (Jenkins/Freckman) (Fig. 1). Per-sample method descriptions, sampling depth, and data provider information are available via figshare^17^. For previously published data, we provide references to the original publications of the respective samples.

### Environmental metadata: soil, climate, topography, vegetation, anthropogenic characteristics

For all sampling locations we provide paired environmental metadata, which can be used to provide insight into the environmental drivers of soil nematode abundance and community composition across spatial scales. To do so, we first prepared a covariate stack of 73 layers, for which we downloaded the covariate layers as geotiff files. Next, all layers were resampled and reprojected to a unified pixel grid in EPSG:4326 (WGS84) at 30 arc-seconds resolution. Layers with a higher original pixel resolution were downsampled using a mean aggregation method; layers with a lower original resolution were resampled using simple upsampling (i.e. without interpolation) to align with the higher resolution grid. Next, all layers were converted into a multiband image, i.e. the covariate stack, that was used for pixel sampling.

**Fig. 2:**
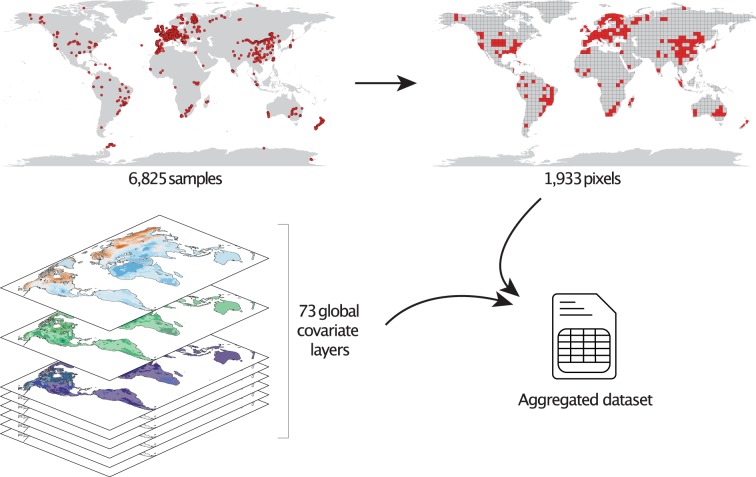
Data processing approach. 6,825 georeferenced samples are included in the raw dataset. These sampling locations represent 1,933 unique 30 arc-seconds pixels (~1 km at the equator), or 1,895 pixels excluding locations falling off the covariate grid. To gain mechanistic insights and discern the major environmental drivers of nematode abundance, these pixels were sampled across 73 global covariate layers.

To prepare the dataset for this purpose, we first need to match the resolution of the dataset to that of the global covariate layer stack that contains the environmental metadata: 30 arc-seconds, which corresponds to approximately 1-km^2^ at the equator. In this step, we aggregate all data points falling within the same pixel by taking the mean value, resulting in 1,933 unique pixels. We stress that the covariate layer stack has no coverage in Antarctica and therefore the 503 samples located in this region were dropped at the pixel aggregation step. Next, pixel values across the 73 layers were retrieved and stored as a csv file. This dataset is available via figshare^17^. We stress that, as some covariate layers were reprocessed since the publication of the nematode mapping study^6^, there are some slight differences in the sampled covariate data in this updated version. The approach is visualized in Fig. 2.

Full metadata, including descriptions, units, and source information of all global covariate layers are available via figshare^17^. In short, information about soil texture and physiochemical properties was obtained from SoilGrids^18^, limited to the upper soil layer (top 15 cm). Climate information was obtained from WorldClim^19^ (version 2), which includes climate data averaged across 1970–2000 (http://www.worldclim.org/). Plant productivity data (i.e. EVI, NDVI, Gpp, Npp) and spectral reflectance data were obtained from Google Earth Engine (https://developers.google.com/earth-engine/datasets/). Aridity index and potential evapotranspiration layers were obtained from CGIAR^20^ (version 1) (http://www.cgiar-csi.org/data/global-aridity-and-pet-database). Anthropogenic information (i.e. human development, population density) was obtained from WCS^21^ (http://wcshumanfootprint.org) and from Tuanmu and Jetz^22^. Aboveground biomass data was obtained from CDIAC^23^ (https://cdiac.ess-dive.lbl.gov/epubs/ndp/global_carbon/carbon_documentation.html). Radiation data was obtained from CliMond^24^ (https://www.climond.org/BioclimRegistry.aspx#BioclimFAQ). WWF Ecoregion classifications were used to categorize sampling locations into biomes (https://www.worldwildlife.org/biome-categories/terrestrial-ecoregions).

## Data Records

All data are available via figshare^17^. Raw nematode abundance data (6,825 samples) are available as a csv file: “nematode_full_dataset_wBiome.csv”. Sample IDs 20001–20066 are samples not present in our related work^6^. Abundance data aggregated into 30 Arc-seconds pixels (1,933 unique locations), combined with environmental covariate data are available as a csv file: “nematode_abundance_aggregated_wCovar.csv”. Full metadata, including descriptions, units, and source information, of all global covariate layers are available as a csv file: “metadata.csv”.

## Technical Validation

**Fig. 3:**
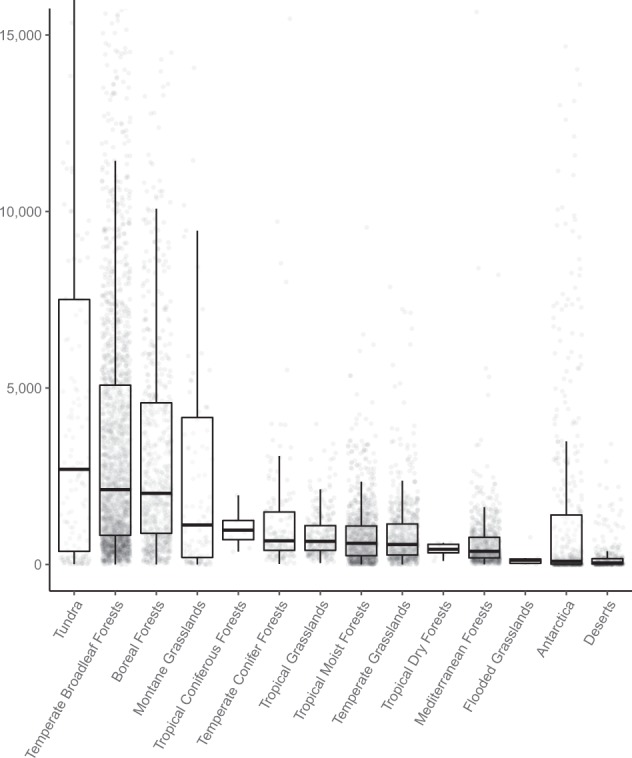
Nematode communities vary across biomes. The median and interquartile range of nematode abundances (n = 6,825) per biome from all continents.

Soil nematode abundances are highly variable within and across terrestrial biomes^6^. On average, the number of nematodes per 100 g dry soil is in the few hundred – few thousand range (median = 859, mean = 2,671), although the highest recorded abundances exceed 20,000 nematodes per 100 g dry soil. Across biomes, bacterivores are the most abundant trophic group and predatory nematodes the least abundant (Table 1). Overall, the highest abundances are observed in tundra (median = 2,695 nematodes per 100 g dry soil), temperate broadleaf forest (median = 2,119) and in boreal forest (median = 2,016) soils. The lowest abundances are observed in Mediterranean forest (median = 374), flooded grasslands (median = 124), Antarctic (median = 89) and hot desert (median = 44) soils (Fig. 3, Table 2). We stress that these numbers slightly differ from the values reported in our accompanying paper^6^, where we reported the aggregated pixel median values.

**Table 1:** Mean and median nematode abundances, per trophic group.

Group	mean	median	n
Bacterivores	1052	250	6788
Fungivores	438	84	6782
Herbivores	656	171	6784
Omnivores	325	41	6787
Predators	119	6	6706
Total_Number	2653	857	6825

Values are reported as the number of nematodes per 100 g dry soil.

**Table 2:** Mean and median nematode abundances, per biome.

Biome	mean	median	n
Tundra	7298	2695	148
Temperate Broadleaf Forests	4465	2120	2175
Boreal Forests	3959	2016	669
Montane Grasslands	6096	1120	116
Tropical Coniferous Forests	1000	970	8
Temperate Conifer Forests	1800	670	158
Tropical Grasslands	863	657	272
Tropical Moist Forests	914	601	968
Temperate Grasslands	945	565	627
Tropical Dry Forests	430	431	11
Mediterranean Forests	619	374	704
Flooded Grasslands	183	124	7
Antarctica	2245	89	503
Deserts	193	44	361

Values are reported as the total number of nematodes per 100 g dry soil.

As with any global ecological dataset, combining data from many researchers across the world, there is inherent variation in the data. Also, the different nematode extraction methods may vary in their efficiencies^25,26^. This underscores the need for large datasets for global scale analyses of ecological patterns. When a sufficiently large sample size allows to detect strong patterns through this statistical noise, we can be confident that a biological pattern exists^6^. As a consequence, there may be limitations to the use of the dataset at finer scales. Yet, by subsetting the dataset by extraction method or region, for example, it can serve as a starting point for local scale studies.

### Environmental representativeness of the dataset

**Fig. 4:**
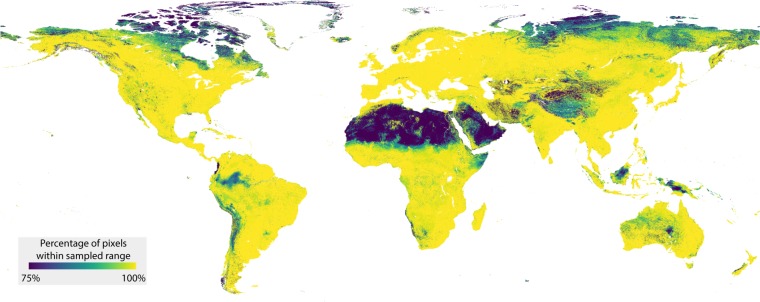
Environmental representativeness of the dataset. The sampled locations represent a wide range of environmental conditions. For illustrative purposes, ten environmental variables were chosen from the full set of 73.

**Fig. 5:**
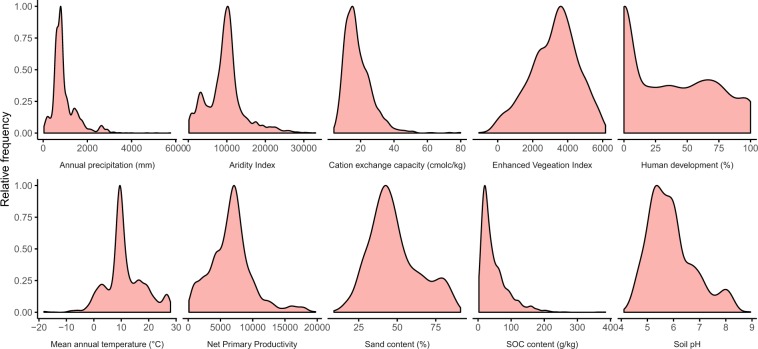
Assessment of the representativeness of the dataset in multivariate environmental space. The map displays the percentage of pixels that fall within the convex hulls of the first 17 principal component spaces (collectively covering >90% of the sample space variation).

To evaluate the comprehensiveness of the dataset, we explored the environmental conditions that the sampling locations represent. Across individual environmental variables, the samples represent a wide range of environmental conditions (Fig. 4). To gain spatial insight into the environmental representativeness of the dataset, information that is important when comparing observations across spatial scales, we evaluated how the multidimensional environmental space covered by the dataset compares to the global environmental space. To do so, we used a similar approach as in our previous work^6^. First, we set out to reduce the computational load, as exploring the full stack of 73 global environmental covariate layers across ~210 million terrestrial pixels would require exorbitantly large computing power. To this end, we transformed the set of global environmental covariate layers into Principal Component (PC) space. We reduced the number of selected PCs to 17, collectively explaining more than 90% of variation. Next, we assessed the proportion of the world’s terrestrial pixels falling within convex hulls of the 136 bivariate combinations of the 17 PCs. The resulting map provides a spatially-explicit depiction of the representativeness of the dataset, showing that the majority of the terrestrial pixels fall within these convex hulls, with most of the outliers existing in arid regions such as the Sahara and Arabian Deserts, and in sub-arctic regions such as the far north of Canada and Russia (Fig. 5).

## Data Availability

Code is available via https://github.com/hooge104/2020_global_nematode_dataset.

## References

[1] Crowther TW (2019). The global soil community and its influence on biogeochemistry. Science.

[2] Crowther TW, Boddy L, Jones TH (2011). Species-specific effects of soil fauna on fungal foraging and decomposition. Oecologia.

[3] Ferris H (2010). Contribution of nematodes to the structure and function of the soil food web. J. Nemat..

[4] Ingham RE, Trofymow JA, Ingham ER, Coleman DC (1985). Interactions of bacteria, fungi, and their nematode grazers: effects on nutrient cycling and plant growth. Ecol. Monogr..

[5] Neher D (2001). Role of nematodes in soil health and their use as indicators. J. Nemat..

[6] van den Hoogen J (2019). Soil nematode abundance and functional group composition at a global scale. Nature.

[7] Bahram M (2018). Structure and function of the global topsoil microbiome. Nature.

[8] Phillips HRP (2019). Global distribution of earthworm diversity. Science.

[9] Yeates GW, Bongers T, De Goede RGM, Freckman DW, Georgieva SS (1993). Feeding habits in soil nematode families and genera–an outline for soil ecologists. J. Nematol..

[10] Baermann G (1917). Eine einfache methode zur auffindung von *Ancylostomum* (Nematoden) larven in erdproben. Geneeskd Tijdschr Ned Indie.

[11] Jenkins WR (1964). A rapid centrifugal-flotation technique for separating nematodes from soil. Plant Dis. Rep..

[12] Freckman DW, Virginia RA (1997). Low-diversity antarctic soil nematode communities: distribution and response to disturbance. Ecology.

[13] Flegg JJM (1967). Extraction of *Xiphinema* and *Longidorus* species from soil by a modification of Cobb’s decanting and sieving technique. Ann. Appl. Biol..

[14] Oostenbrink, M. Estimating nematode populations by some selected methods. *Nematology* **6**, 85–102 (1960).

[15] Whitehead AG, Hemming JR (1965). A comparison of some quantitative methods of extracting small vermiform nematodes from soil. Ann.Appl. Biol..

[16] Seinhorst J, others (1965). The relation between nematode density and damage to plants. Nematologica.

[17] van den Hoogen J (2020). figshare.

[18] Hengl T (2017). SoilGrids250m: Global gridded soil information based on machine learning. PLoS ONE.

[19] Fick SE, Hijmans RJ (2017). WorldClim 2: new 1-km spatial resolution climate surfaces for global land areas. Int. J. Climatol.

[20] Zomer RJ, Trabucco A, Bossio DA, Verchot LV (2008). Climate change mitigation: A spatial analysis of global land suitability for clean development mechanism afforestation and reforestation. Agr. Ecosyst. Environ..

[21] Venter O (2016). Global terrestrial human footprint maps for 1993 and 2009. Sci. Data.

[22] Tuanmu M-N, Jetz W (2014). A global 1-km consensus land-cover product for biodiversity and ecosystem modelling: Consensus land cover. Global Ecol. Biogeogr..

[23] Ruesch, A. & Gibbs, H. K. New IPCC Tier-1 global biomass carbon map for the year 2000, https://cdiac.ess-dive.lbl.gov (2008).

[24] Kriticos DJ (2012). CliMond: global high-resolution historical and future scenario climate surfaces for bioclimatic modelling: CliMond: climate surfaces for bioclimatic modelling. Methods Ecol. Evol..

[25] De Goede RG, Verschoor B (2000). The nematode extraction efficiency of the Oostenbrink elutriator-cottonwool filter method with special reference to nematode body size and life Strategy. Nematology.

[26] Cesarz S, Schulz AE, Beugnon R, Eisenhauer N (2019). Testing soil nematode extraction efficiency using different variations of the Baermann funnel method. Soil Org..

